# Assessment of squamous cell carcinoma of the floor of the mouth with magnetic resonance imaging

**DOI:** 10.1007/s11604-021-01161-1

**Published:** 2021-07-07

**Authors:** Akira Baba, Kazuhiko Hashimoto, Hirofumi Kuno, Koichi Masuda, Satoshi Matsushima, Hideomi Yamauchi, Koshi Ikeda, Masae Yamazaki, Suzuki Taiki, Satoru Ogane, Ryo Kurokawa, Yoshiaki Ota, Takeshi Nomura, Hiroya Ojiri

**Affiliations:** 1 Department of Radiology, Jikei University School of Medicine, 3-25-8 Nishi-Shimbashi, Minato-ku, Tokyo 105-8461 Japan; 2 Department of Radiology, University of Michigan, 1500 E Medical Center Dr, UH B2, Ann Arbor, Michigan 48109 USA; 3 Department of Pathology and Laboratory Medicine, Tokyo Dental College Ichikawa General Hospital, 5-11-13 Sugano, Ichikawa-shi, Chiba 272-8513 Japan; 4 Department of Diagnostic Radiology, National Cancer Center Hospital East, 6-5-1 Kashiwanoha, Kashiwa-shi, Chiba 277-8577 Japan; 5 Department of Radiology, Tokyo Dental College Ichikawa General Hospital, 5-11-13 Sugano, Ichikawa-shi, Chiba 272-8513 Japan; 6 Oral Cancer Center, Tokyo Dental College, 5-11-13 Sugano, Ichikawa-shi, Chiba 272-8513 Japan; 7 Department of Radiology, Graduate School of Medicine, The University of Tokyo, 7-3-1 Hongo, Bunkyo-ku, Tokyo 113-8655 Japan

**Keywords:** Mouth floor, Carcinoma, Squamous cell, Magnetic resonance imaging, Neoplasm invasiveness

## Abstract

**Purpose:**

We aimed to use magnetic resonance imaging (MRI) to determine the relationship between the pathological depth of invasion (DOI), undetectability, and tumor thickness of squamous cell carcinoma of the floor of the mouth.

**Materials and methods:**

We retrospectively evaluated the relationship between pathological DOI and MRI detectability, as well as the relationship between pathological DOI and tumor thickness on coronal fat-suppressed contrast-enhanced T1-weighted imaging or coronal T2-weighted imaging.

**Results:**

We analyzed 30 patients with squamous cell carcinoma of the floor of the mouth; MRI revealed that the pathological DOI of the 11 undetectable lesions (median 2 mm) was smaller than that of the 19 detectable lesions (median 14 mm) (*p* < 0.001), and the cut-off value was 3 mm (sensitivity, 0.84; specificity, 0.91; area under the curve, 0.89). Tumor thickness on coronal fat-suppressed contrast-enhanced T1-weighted imaging was assessed in all 19 detectable lesions; however, tumor thickness on coronal T2-weighted imaging could not be assessed in eight cases. Tumor thickness on coronal fat-suppressed contrast-enhanced T1-weighted imaging was found to be significantly associated with the pathological DOI.

**Conclusions:**

Undetectability on MRI indicates superficial lesions with a pathological DOI value that is less than 3 mm. In detectable lesions, tumor thickness on coronal fat-suppressed contrast-enhanced T1-weighted imaging is associated with pathological DOI.

## Introduction

Squamous cell carcinoma is the most common oral cancer, with the tongue being the most commonly affected site, followed by the mandibular gingiva and the floor of the mouth [1]. In the 8th edition of the Cancer Staging Manual by the American Joint Committee on Cancer (AJCC), depth of invasion (DOI) has been added to the T-staging criteria for oral cancer [2]. This is because pathological DOI is strongly associated with cervical lymph node metastasis, which is the most influential negative prognostic. An association between pathological DOI and prognosis has been reported for oral cancers, including floor of the mouth cancer [3, 4]. In clinical practice, MRI is widely used for the T-staging of oral cancer due to its ability to provide a detailed visualization of the extent of the tumor. However, the cancer staging manual does not describe how to estimate the pathological DOI before treatment by radiological assessment [2]. Thus, it is important to standardize the pre-treatment estimation of DOI by imaging.

Previous studies focusing on tongue cancer have reported a relationship between DOI as measured on MRI or contrast-enhanced computed tomography and pathological DOI [5–9]. Moreover, it was reported that the undetectability of tongue cancer on MRI was associated with low values of pathological DOI [5, 6].

Notably, there has been no reports regarding MRI measures that relate with pathological DOI or any MRI findings to estimate pathological DOI in the case of floor of the mouth cancer. In addition, undetectability on MRI may suggest superficial low-volume lesions, i.e., lesions with low pathological DOI such as floor of the mouth cancer as well as tongue cancer. Clinically, it is not practical to measure DOI of floor of mouth cancer using MRI, as the adjacent mucosa cannot be clearly identified, and it seems acceptable to use tumor thickness as a substitute for the DOI. Thus, we hypothesized that MRI measurements and MRI undetectability might be associated with pathological DOI of floor of the mouth cancer and could be used for its estimation. The purpose of this study was to investigate the relationship between MRI measurements and MRI detectability, and pathological DOI.

## Materials and methods

### Patients

We retrospectively evaluated consecutive patients who underwent radical surgery for primary squamous cell carcinoma of the floor of the mouth, and pre-treatment MRI, including T2-weighted and contrast-enhanced fat-suppressed T1-weighted imaging between April 2009 and August 2020. We excluded patients with MRIs that were difficult to assess due to imaging artifacts. This retrospective study was approved by the institutional review board and ethics committee. Since this was a retrospective study, the requirement for informed consent was waived.

### Evaluation of MRI scans

Two board-certified radiologists retrospectively and independently evaluated MRI tumor detectability and thickness of floor of the mouth cancer lesions in all patients, independently. The average value of the results obtained by the radiologists was used in the evaluation of tumor thickness on MRI. We determined the location of the tumor and the direction of tumor thickness on MRI based on coronal planes. In the evaluation of MR detectability, if the two observers reached the same result, that result was used; however, when the results differed, the final result was decided by consensus. We used a Picture Archiving and Communication System (Synapse Viewer; Fuji Medical Systems, Tokyo, Japan) to view and evaluate the scans digitally.

**Fig. 1:**
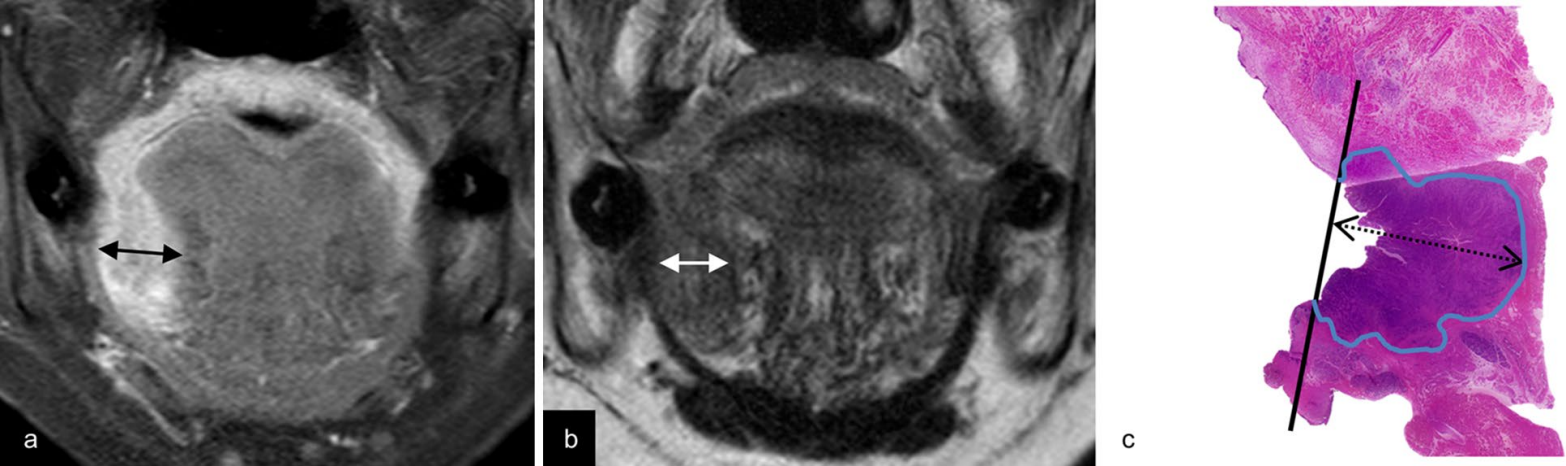
Estimation of tumor thickness on CET1WI, tumor thickness on T2WI, and pathological DOI. A 57-year-old female with a right-sided squamous cell carcinoma of the floor of the mouth. Coronal fat-suppressed contrast-enhanced T1-weighted image (**a**) and coronal T2-weighted image (**b**) reveal a right-sided carcinoma in the floor of the mouth. Tumor thickness on CET1WI and tumor thickness on T2WI (two-directional arrow) are measured from the surface to the deepest aspect of the tumor. An image of a hematoxylin and eosin-stained pathological specimen (**c**) shows a lesion with pathological DOI (two-directional dotted arrow) measured from the horizontal reference line (solid line), connecting the basement membrane of the adjacent normal squamous mucosa, to the deepest aspect of the tumor. Tumor thickness on CET1WI is 13.7 mm, tumor thickness on T2WI is 13 mm, and pathological DOI is 14 mm. Note—DOI, depth of invasion; CET1WI, contrast-enhanced T1-weighted imaging; T2WI, T2-weighted imaging

**Fig. 2:**
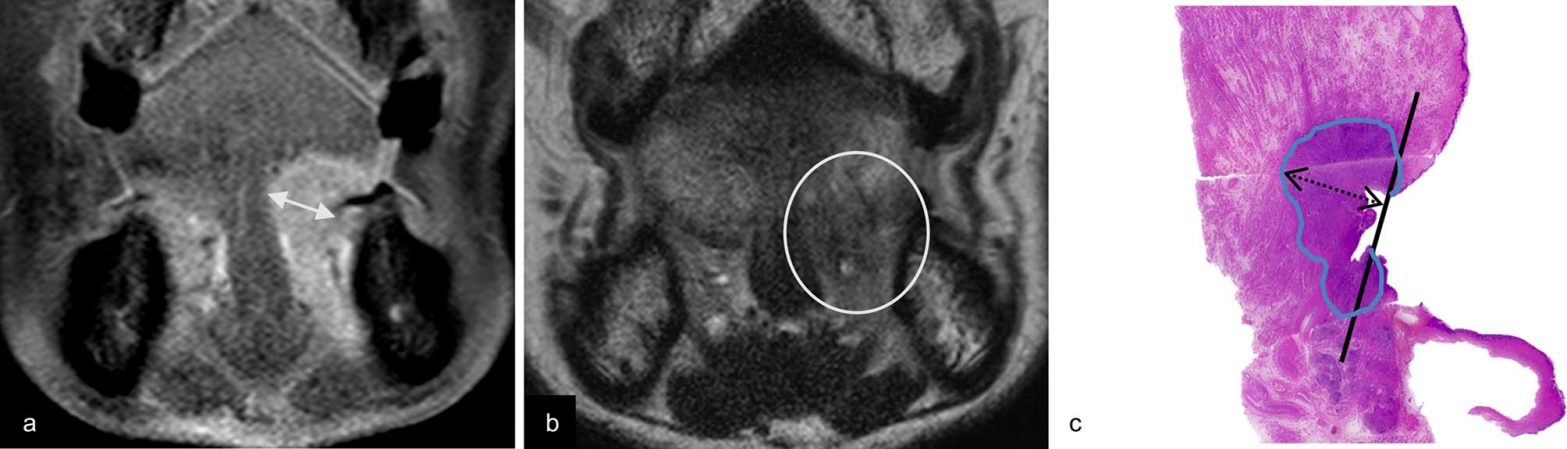
Estimation of tumor thickness on CET1WI, tumor thickness on T2WI, and pathological DOI. An 83-year-old male with a left-sided squamous cell carcinoma of the floor of the mouth. Coronal fat-suppressed contrast-enhanced T1-weighted image (**a**) reveals a left-sided carcinoma in the floor of the mouth. Tumor thickness on CET1WI (two-directional arrow) is measured. Coronal T2-weighted image (**b**) shows abnormal lesion as a faint low signal intensity structure (within circle), however, an obvious mass lesion in the left oral floor region is not observed. Tumor thickness on T2WI is not able to be evaluated due to absence of clear contrast between the tumor and adjacent tissue. An image of a hematoxylin and eosin-stained pathological specimen (**c**) shows a lesion with pathological DOI (two-directional dotted arrow). Tumor thickness on CET1WI is 12.1 mm, and pathological DOI is 11 mm. Note—DOI, depth of invasion; CET1WI, contrast-enhanced T1-weighted imaging; T2WI, T2-weighted imaging

**Fig. 3:**
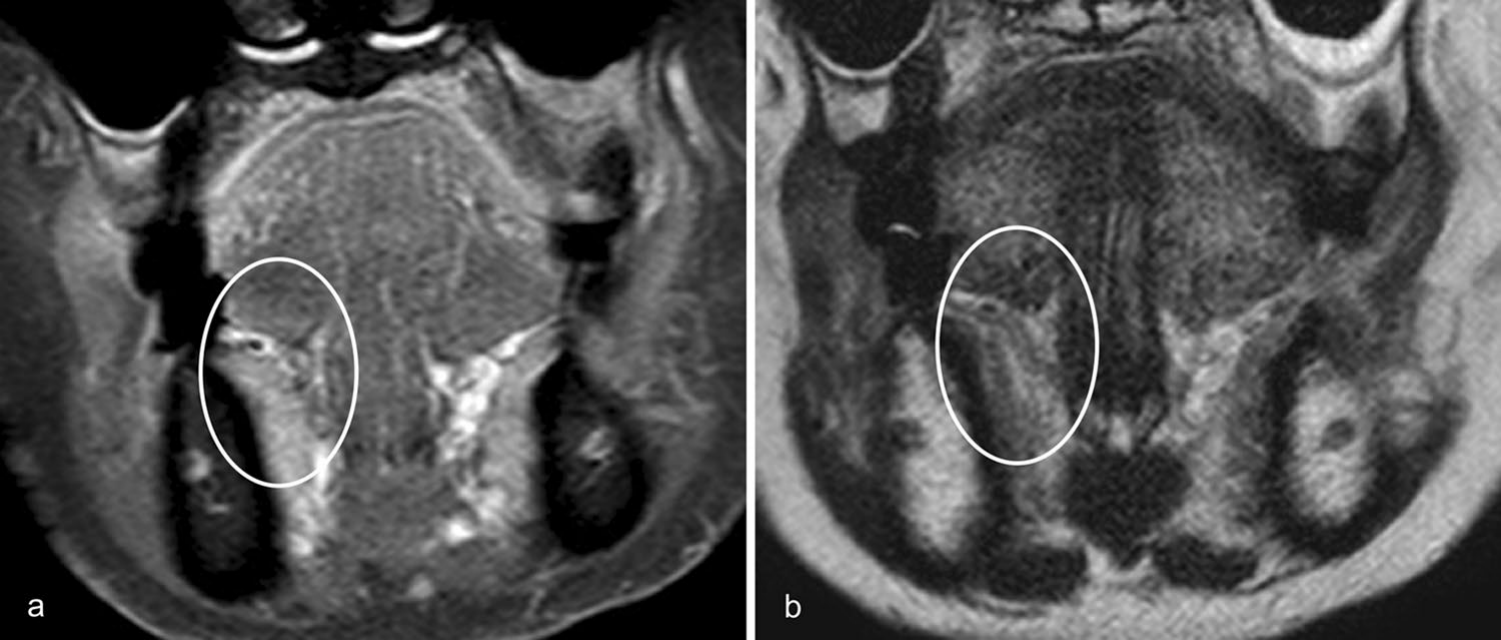
Undetectability of floor of the mouth cancer on MRI. An 88-year-old female with a right-sided squamous cell carcinoma of the floor of mouth. Both coronal fat-suppressed contrast-enhanced T1-weighted image (**a**) and coronal T2-weighted image (**b**) do not reveal any lesion (undetectable lesion) on the right floor of the mouth (within the circle) with clinically and pathologically proven right-sided squamous cell carcinoma of the floor of mouth

We substituted the DOI with tumor thickness for MRI measurement purposes. Tumor thickness was measured as the maximum shortest diameter of the lesion in the slice where the lesion was largest, using coronal fat-suppressed contrast-enhanced T1-weighted imaging (CET1WI) (Figs. 1a, 2a) and coronal T2-weighted imaging (T2WI) (Fig. 1b). Lesions were considered as detectable lesions when they were identified on T2WI or CET1WI, and as undetectable lesions when lesions were not identified on both MRI sequences (Fig. 3).

MRI were performed on a 1.5-T system (Achieva; Philips Medical Systems) using a maximum gradient field strength of 33 mT/m and a 2-ch Flex S coil and on 3.0-T system (Ingenia; Philips Medical Systems) using a maximum gradient field strength of 45 mT/m and a Head Neck coil. All patients were examined in the supine position. All fields-of-view were set to the maxillofacial region. Coronal T2WI was obtained using the following parameters: TR/TE, 3000–4300 ms/84–90 ms; flip angle, 90°; field-of-view, 15–18 × 15–18 cm; matrix size, 288 × 214–230; slice thickness, 3.5–4 mm; gap, 0.3–0.4 mm; NEX, 1–2. Coronal CET1WI was obtained by using the following parameters: TR/TE, 550 ms/10 ms; flip angle, 90°; field-of-view, 15 × 15 cm; matrix size, 320 × 256; slice thickness, 3.5 mm; gap, 0.3 mm; NEX, 2 and coronal CET1WI (mDIXON water images) was obtained using the following parameters: TR/TE1/TE2, (shortest) 6 ms/(shortest) 2 ms/(shortest) 3 ms;f lip angle, 15°; field-of-view, 20 × 20 cm; matrix size, 192 × 190; slice thickness, 1.1 mm; NEX, 1. Biopsies were routinely performed approximately a week before surgery in our institution; thus, the effect of the biopsy could not reflect in MRI performed prior to the biopsy.

### Pathological evaluation

Tissue samples from surgical specimens resected in the coronal (16 cases) or sagittal (14 cases) plane were subjected to histopathological analysis. The specimen was stretched with a pin on a rubber plate and fixed with 10% formalin. Thus, the shrinkage of the specimen due to fixation could be suppressed. Next, they were sectioned, and stained with hematoxylin and eosin. The sliced specimens were evaluated by an oral pathologist specializing in oral cancer pathology. Pathological DOI was measured according to the AJCC Cancer Staging System, 8th edition [2], including descriptions that “DOI is measured by first finding the ‘horizon’ of the basement membrane of the adjacent squamous mucosa. A perpendicular ‘plumb line’ is established from this horizon to the deepest point of tumor invasion.” (Figs. 1c, 2c).

### Neck node positivity

In clinical N0 cases with more than 2 years of follow-up, we investigated 2 year potential lymph node metastases (defined as pathologically positive for lymph node metastases at the time of operation or appearance of lymph node metastases within 2 years) for potential neck node positivity. Additionally, the 2 year potential lymph node metastases were compared between undetectable and detectable lesions.

### Statistical analysis

The Shapiro–Wilk test showed that all data were not normally distributed. The Mann–Whitney *U* test was used to compare the pathological DOI between the undetectable and detectable lesions, and to evaluate differences between tumor thickness on CET1WI and pathological DOI on 1.5 T and 3 T MRI. We calculated the cut-off value of the pathological DOI between undetectable lesion and detectable lesion using the Youden index from the receiver operating characteristic (ROC) curve. The Fisher’s exact test was used to compare the 2 year potential rate of neck lymph node metastasis between undetectable and detectable lesions. Interobserver agreement in evaluation of MRI detectability was assessed by weighted kappa statistics. A value of 0–0.20 indicated poor agreement, 0.21–0.40 indicated fair agreement, 0.41–0.60 indicated moderate agreement, 0.61–0.80 indicated good agreement, and 0.81–1.00 indicated very good agreement. A Bland Altman plot was used to evaluate the relationship between pathological DOI and tumor thickness on CET1WI, as well as T2WI. The intraclass correlation coefficients (ICCs) based on two-way random effects models were also calculated. Using common criteria [10], measurement reliability was classified as poor (ICC < 0.40), fair (ICC = 0.40–0.59), good (ICC = 0.60–0.74), or excellent (ICC > 0.75). A *p* value of < 0.05 was considered to indicate statistical significance. All statistical analyses were performed using R version 3.6.1 (R Foundation for Statistical Computing, Vienna, Austria).

## Results

**Fig. 4:**
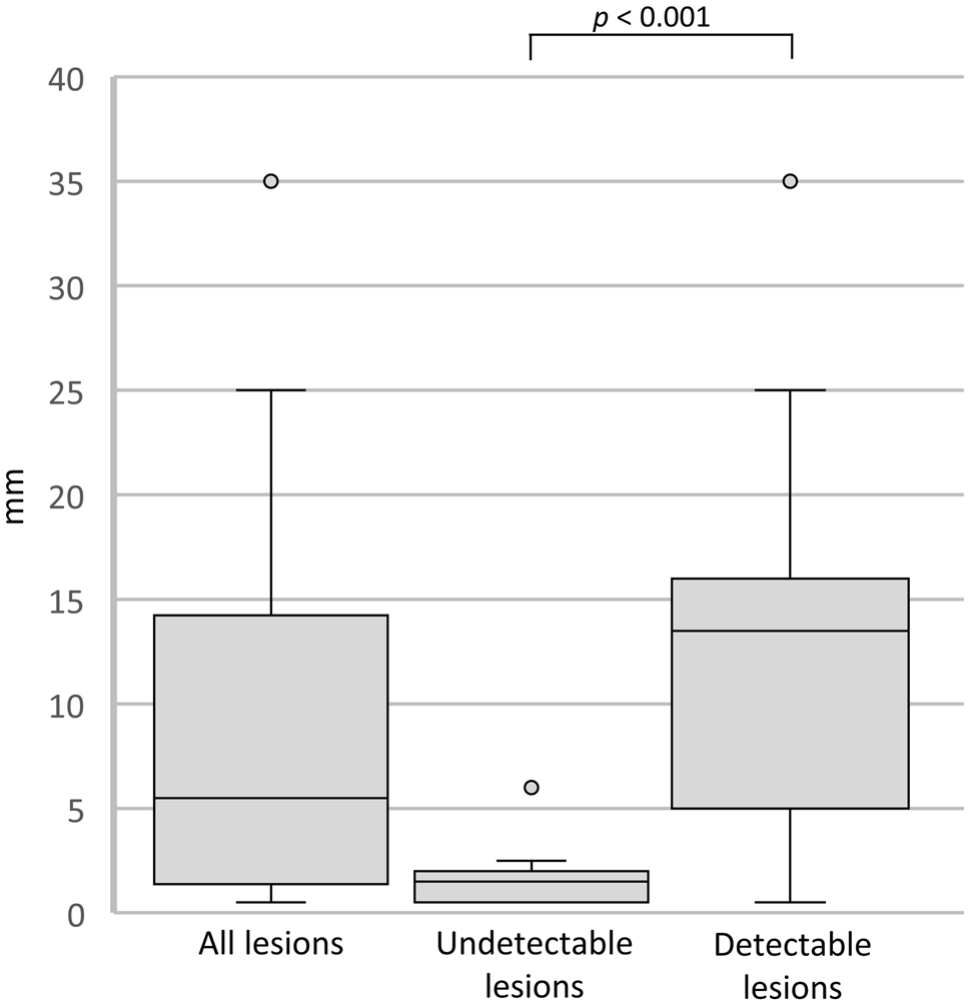
Box-and-whisker plots of pathological DOI in all lesions (undetectable and detectable). Note—DOI, depth of invasion

In total, we evaluated 30 patients with squamous cell carcinoma of the floor of the mouth [18 males and 12 females; age: range 45–83 years; average ± standard deviation (SD), 67 ± 10.3 years]. The T-staging category ranged from T1 to T4a (T1: 10 cases, T2: 8 cases, T3: 10 cases, T4a: 2 cases) and the N-staging ranged from N0 to N3b (N0: 25 cases, N2b: 4 cases, N3b: 1 cases) as classified by the AJCC Cancer Staging Manual, 8th edition [2]. The median pathological DOI was 5 mm (range 1–35; IQR 2–14) (Fig. 4). Elective neck dissection was performed in 14 cases, and 7 cases were found to be neck node metastasis positive. In 21 cases with clinical N0 stage, 2 year potential lymph node metastasis was detected in 7 cases.

**Fig. 5:**
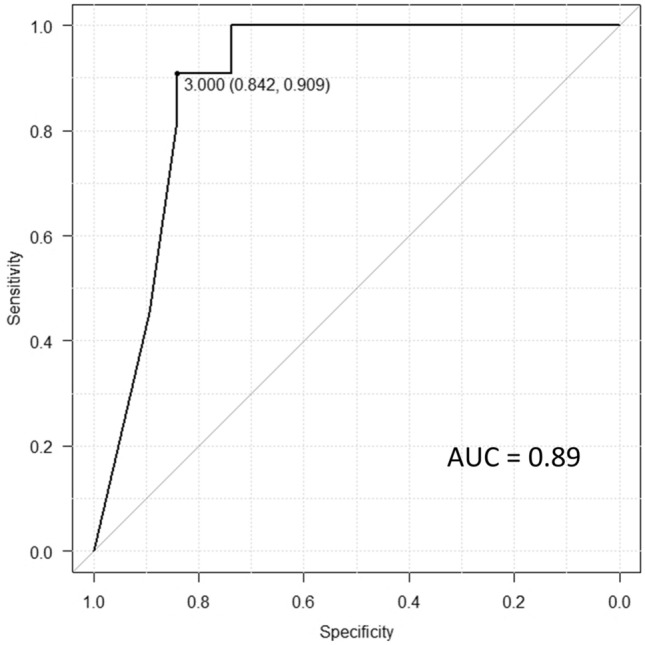
ROC curve of the cut-off of pathological DOI between the undetectable and detectable lesions. Note—ROC, receiver operating characteristic; DOI, depth of invasion; AUC, area under curve

Undetectable lesions were determined in 11 cases (36.7%) and detectable lesions in 19 cases (63.3%). In univariate analyses, pathological DOI of undetectable lesions (median 2 mm; range 1–6 mm; IQR 1–2) was smaller than that of detectable lesions (median 14 mm; range 1–35 mm; IQR 6–16) (*p* < 0.001) (Fig. 4). The cut-off value of pathological DOI between undetectable lesion and detectable lesion was 3 mm [sensitivity, 0.84; specificity, 0.91; area under the curve, 0.89 (95% confidence interval 0.77–1.00)] (Fig. 5). The 2 year potential lymph node metastases rate of undetectable lesions was lower than that of detectable lesions (1/8, 13% vs 6/13, 46%), however, the difference was not significant (*p* = 0.17). Interobserver agreement in the evaluation of detectability on MRI was good [kappa-value = 0.78 (95% confidence interval 0.54–1.00)].

The difference between tumor thickness on CET1WI and pathological DOI with 1.5 T MRI in 13 cases was 3.3 mm (range − 12.7–12.2; IQR 0.2–5.1), and that with 3 T MRI in 7 cases was 3.9 mm (range − 3.3–9.2; IQR 3.3–6.8); there was no significant difference between the two MRI modalities (*p* = 0.588).

Tumor thickness on CET1WI was adequately evaluated in all 19 cases with detectable lesion. Tumor thickness on T2WI could be assessed in 11 of 19 cases (58%) because of the less of clear contrast between the tumor and the adjacent tissue obscured detailed localization (Fig. 2b).

**Fig. 6:**
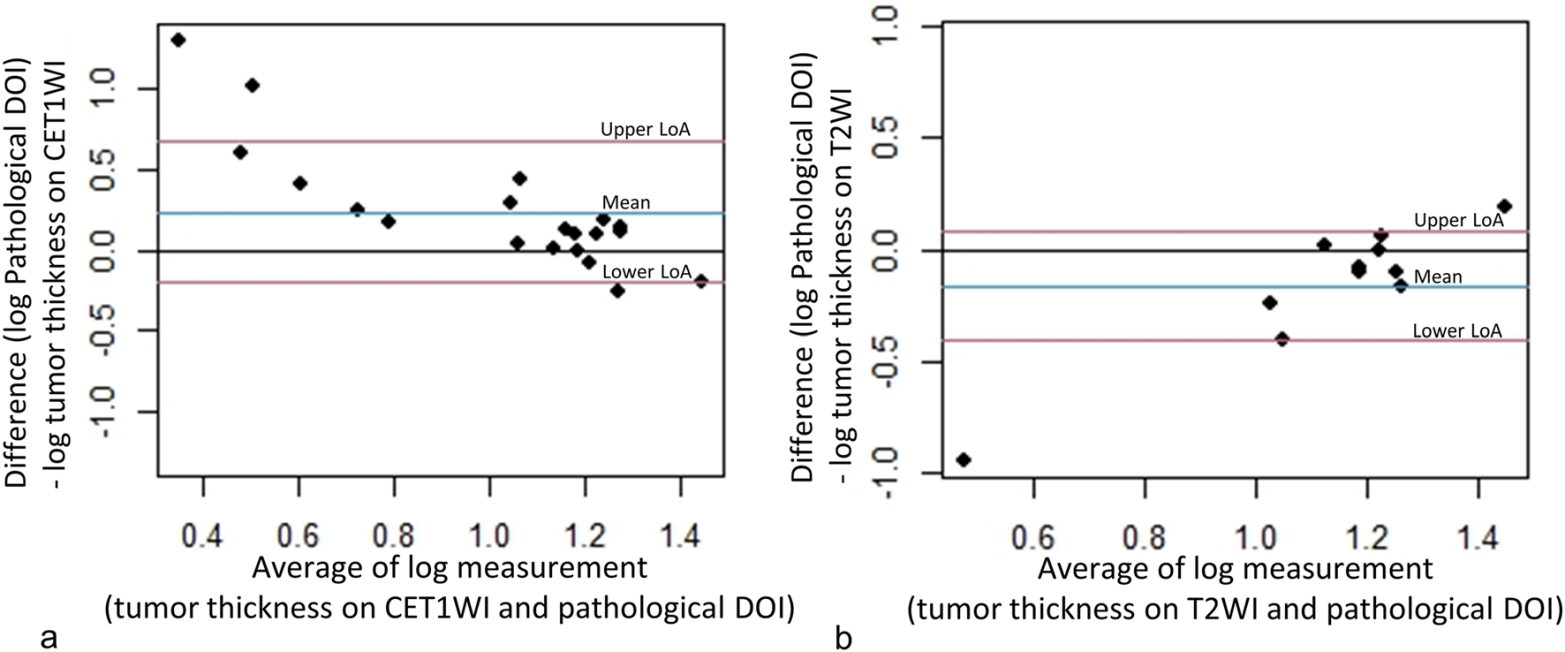
The Bland Altman plot between tumor thickness on CET1WI and pathological DOI (**a**), and between tumor thickness on T2WI and pathological DOI (**b**) after log transformation. Note—DOI, depth of invasion; LoA, limit of agreement; CET1WI, contrast-enhanced T1-weighted imaging; T2WI, T2-weighted imaging

**Fig. 7:**
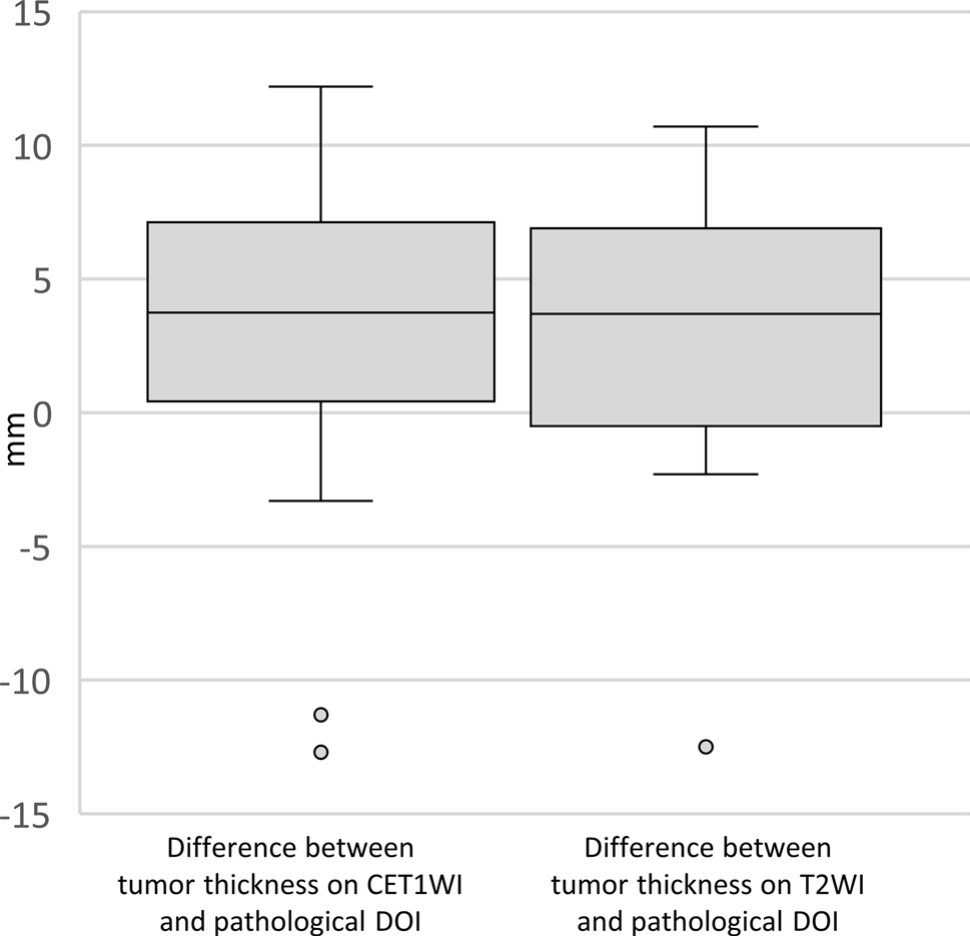
Box-and-whisker plots of the median difference between tumor thickness on CET1WI and pathological DOI, and that between tumor thickness on T2WI and pathological DOI. Note—DOI, depth of invasion; CET1WI, contrast-enhanced T1-weighted imaging; T2WI, T2-weighted imaging

A Bland–Altman plot between tumor thickness on CET1WI and pathological DOI after log transformation showed a relatively significant association (Fig. 6a). Good agreement was observed between tumor thickness on CET1WI and the pathological DOI (ICC = 0.60). A Bland–Altman plot between tumor thickness on T2WI and pathological DOI after log transformation showed a relatively significant association as well (Fig. 6b). The agreement between tumor thickness on T2WI and pathological DOI was fair (ICC = 0.41). The median difference between tumor thickness on CET1WI and pathological DOI was 3.75 mm (range, − 12.7–12.2; IQR 0.88–6.4), and that between tumor thickness on T2WI and pathological DOI was 3.7 mm (range 10.7–12.5; IQR − 0.15–6.5) (Fig. 7).

## Discussion

In the current study, squamous cell carcinoma of the floor of the mouth lesions which were not detected using MRI suggested a relatively superficial lesion with a pathological DOI < 3 mm. The relationship between tumor thickness on CET1WI and pathological DOI was significant and good.

The 8th edition of the AJCC Cancer Staging Manual added a DOI to the criteria for T classification of oral cancer T1 to T4a [2], because pathological DOI is strongly associated with cervical lymph node metastasis, the most important prognostic factor. It is also associated with a 50% reduction in 5 year survival [11, 12]. The pathological DOI cut-off value for determining prognosis is most commonly set at 4 mm, as a previous review demonstrated that a pathological DOI greater than 4 mm strongly predicts neck lymph node metastasis [13]. Therefore, the National Comprehensive Cancer Network (NCCN) recommends neck dissection in cases with a pathological DOI > 4 mm [14]. Thus, pre-treatment estimation of the DOI is very important clinically, although it is problematic that the standard method for pre-treatment estimation of pathological DOI by radiological evaluation has not been established [2].

Previous work has demonstrated undetectability on MRI to be useful for the estimation of pathological DOI in tongue cancer [6]. Tongue cancer that is pathologically proven, yet not detectable on MRI, might be considered a superficial, low-volume lesion. The pathological DOI of tongue cancer undetected on MRI tended to be 3.5 mm or less, and 96% of the lesions had a pathological DOI < 4 mm [6]. In floor of the mouth cancer, as in previous reports on tongue cancer [6], undetectability on MRI is thought to be due to a superficial lesion and thus suggestive of a small DOI (< 3 mm). Neck lymph node metastasis is the most important prognostic factor for oral cancer [15, 16]. Elective neck dissection has higher overall and disease-free survival rates than therapeutic neck dissection in patients with early-stage oral squamous cell carcinoma [17]. Therefore, both the evaluation and decision for elective neck dissection in patients with oral cancer are clinically important. The NCCN recommends elective neck dissection in cases with a DOI > 4 mm [14], as mentioned previously. Since this criterion focuses on tongue cancer, and oral floor cancer has a higher potential neck lymph node-positive rate than tongue cancer [18], its application to oral floor cancer is controversial. However, undetectable lesions in floor of the mouth cancer on MRI may provide a potential justification for avoiding elective neck dissection.

**Fig. 8:**
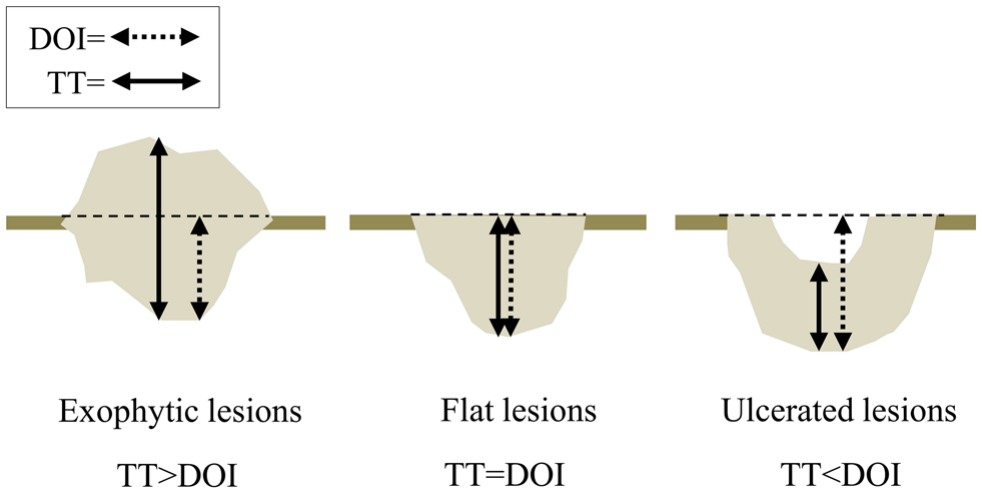
Relationship between tumor thickness and the DOI in different types of lesions. Note—DOI, depth of invasion; TT, tumor thickness

According to previous reports [5–9, 19, 20], measurement of the DOI on MRI is preferable for estimating pathological DOI; however, measurement of the DOI on MRI is difficult in floor of the mouth cancer, because the boundary between the adjacent normal mucosa and the tumor is obscured, and the protruding areas and ulcerative lesions are covered by the tongue. In the present study, we used tumor thickness instead of the DOI as the MRI measurement standard. This is because it is difficult to establish a plumb line in the floor of the mouth for the DOI on MRI, which is relatively easy to measure in tongue cancer, and this may cause some variability in the measurement. The distinction between tumor thickness and the DOI is important because of their varying descriptions in studies on oral cancer [13], and there are substantial differences in their measurement methods (Fig. 8). In clinical practice, it is important to note that the tumor thickness is smaller than the DOI in ulcerative lesions and larger than the DOI in exophytic lesions. The results of the Altman-Bland plot and calculation of ICCs suggest that measuring tumor thickness on CET1WI is a relatively reliable test in assessing pathological DOI in floor of the mouth cancer. In the current study, the T2WI was not always useful for estimating the DOI as it was difficult to assess tumor thickness on T2WI in 42% of the cases of detectable lesions. Since the floor of the mouth region, including the sublingual glands, normally shows a high signal intensity, the difficulty might have been due to the lack of contrast between the carcinoma in the floor of the mouth and adjacent structures with an equally high signal intensity. It should be noted that although T2WI may be less useful for estimating the DOI than CET1WI, it is important to depict the involvement of the surrounding structures such as the extrinsic tongue muscle, as well as the secondary changes associated with movement deep within the oral floor such as submandibular duct dilation. Furthermore, T2WI might be useful for estimating pathologic DOI in patients with contraindications to contrast materials such as renal dysfunction, allergies, and underlying diseases.

One previous study has shown that non-contrast-enhanced T1-weighted imaging is the most suitable method to analyse tumor thickness in tongue cancer [21], thus T1-weighted images may also be useful in the evaluation of floor of the mouth cancer. Coronal non-contrast-enhanced T1-weighted imaging, which we did not perform, might also be useful and needs to be investigated in the future. In addition, the evaluation of 3D sequences on post-contrast T1-weighted imaging may be more useful than conventional contrast-enhanced T1-weighted imaging. Therefore, evaluation limited to 3D sequences will be necessary in the future. Ultrasonography can also aid in assessing tumor extent and the DOI. A strong correlation has been reported between the DOI on ultrasound images and in oral tongue cancer [22]. However, ultrasound may not necessarily be the best examination, because it is sometimes difficult to touch the ultrasound probe to floor of mouth cancer. Therefore, MRI is recommended as a better evaluation modality than ultrasonography in cases of floor of mouth cancer.

In floor of mouth cancer, it is necessary to resect at least the tissue above the hyoid muscle, even if the lesion is superficial and localized to the mucosa. Therefore, the treatment strategy for undetectability lesions should focus on sublingual dissection or resection of the sublingual space. In detectable lesions, invasion of the sublingual space, including the sublingual gland, is often suspected. The treatment strategy for detectable lesions should therefore focus on resection beyond the hyoid muscle to ensure a safe margin. The DOI of floor of mouth cancer may be an important indicator in determining the combination of local resection and flap reconstruction.

There were several limitations to our study. The study was retrospective, included a small number of patients, and involved a single center. Larger studies would be needed to confirm our results. Furthermore, the combined evaluation of 3 T and 1.5 T MRI may reflect differences in imaging quality and detectability among some cases. Another limitation in this study is that the pathology requires evaluation in the coronal and sagittal sections, while MRI is evaluated based on coronal section images only. In our institution, MRI including axial section of T1-weighted and diffusion-weighted images, axial and coronal sections of T2-weighted images, and axial and coronal sections of post-contrast fat suppression T1-weighted images are routinely used during the evaluation for oral cancer. Future prospective studies should additionally include MRI evaluation based on sagittal section images for median oral floor cancer, and the results may differ from the current results in detectability and relationship of MRI with pathologic DOI. Since no association has been observed between the DOI and prognosis in oral floor cancer alone, the current results do not necessarily provide a valid prognostic estimate. Although the relationship between MRI findings and pathology may have differed between edentulous and dentulous cases, there was only one edentulous case in this study, and it was not examined. The importance of using tumor thickness on MRI as a surrogate for the DOI in determining T classification also needs to be investigated in the future, including the clinical outcomes.

## Conclusion

In the case of squamous cell carcinoma of the floor of the mouth, undetectability on MRI suggests a superficial lesion with a pathological DOI < 3 mm. In detectable lesions, a clinically important association existed between tumor thickness on CET1WI and pathological DOI. For patients with contraindications to contrast materials, tumor thickness on T2WI may also be useful for the estimation of pathological DOI.
